# Supplementary material for: A Comprehensive Comparative Analysis and Phylogenetic Investigation of the Chloroplast Genome Sequences in Four Astragalus Species
Source: Curr Issues Mol Biol. 2025 Nov 25;47(12):978. doi: 10.3390/cimb47120978 (PMC12731972; doi:10.3390/cimb47120978)
Supplement: Supplementary file 1 [file cimb-47-00978-s001.zip › Supplementary Figure S1-S2.pdf]

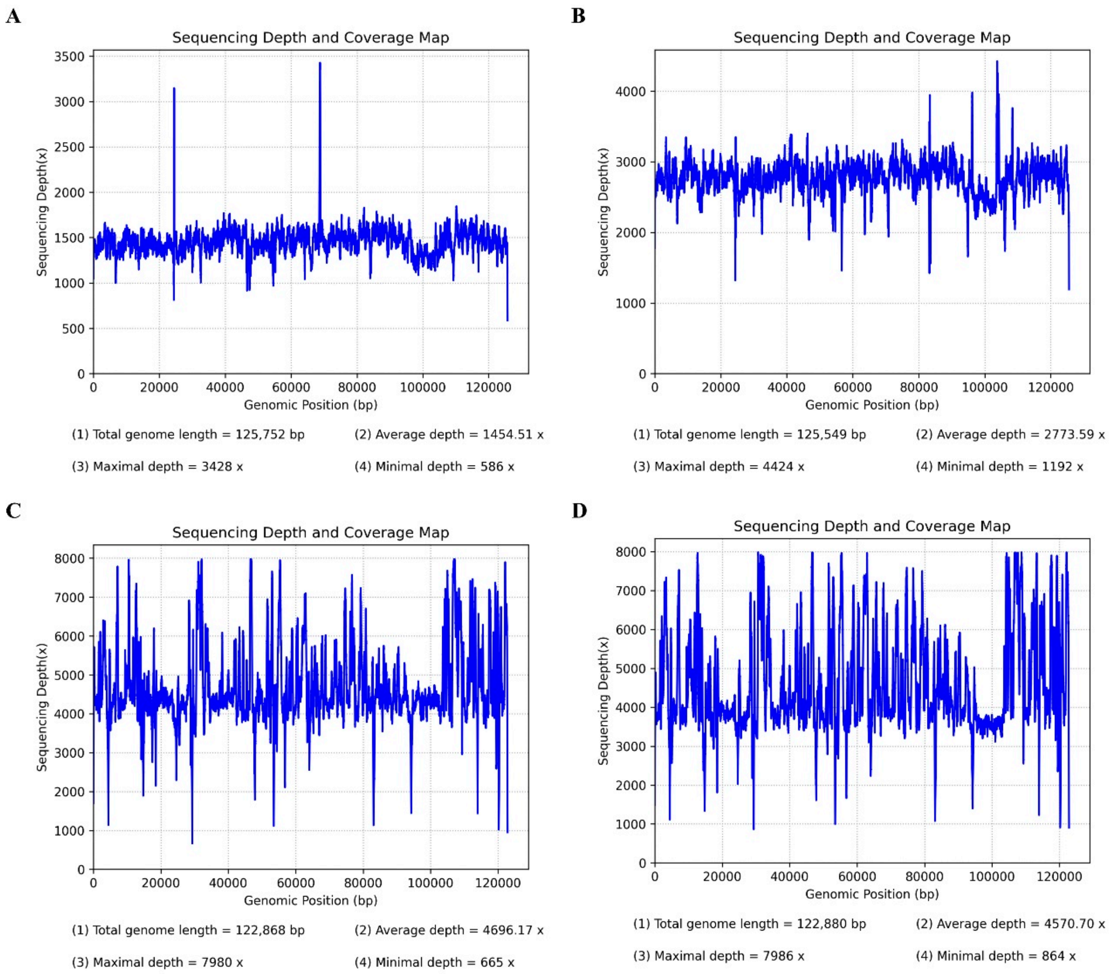

Figure S1 The depth of assemblies about *A. yunnanensis* (A), *A. yunnanensis* subsp. *incanus* (B), *A. polycladus* (C) and *A. polycladus* var. *nigrescens* (D).

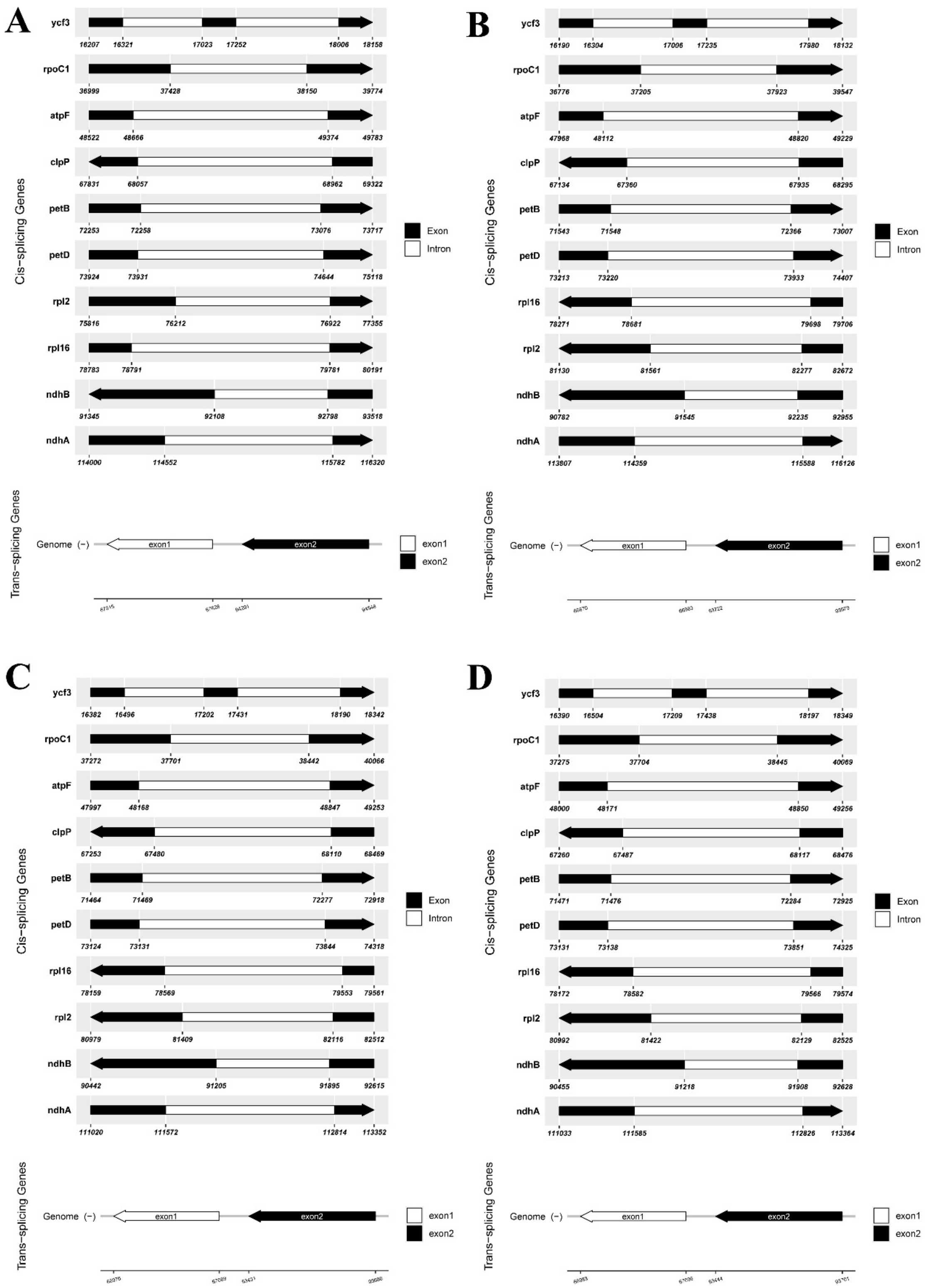

Figure S2 The map of the cis-splicing genes and trans-splicing gene *rps12* in the *A. yunnanensis* (A), *A. yunnanensis* subsp. *incanus* (B), *A. polycladus* (C) and *A. polycladus* var. *nigrescens* (D).
